# Valedictory

**Published:** 1868-04

**Authors:** 


### EDITORIAL DEPARTMENT.

*Valedictory.*—In taking leave of our readers at the close of our new volume, we are happy to be able to congratulate them upon our success This has been far beyond our most sanguine expectations. Our subscription list has steadily increased, and our advertising columns have been well filled without encroaching at all upon the space devoted to reading matter. Of this we have given our subscribers more than we promised in our prospectus.

To all our patrons we return our thanks. We have honestly endeavored to give all their *quid pro quo.* To our contributors we desire to acknowledge a special indebtedness. They have enabled us to give our readers a very large amount of original matter both interesting and profitable to the profession. We ask their further assistance in the second volume of the new series, which will commence with the May number. It is our determination to make this Journal worthy of the support of the profession everywhere. Its columns are open to all who have anything to suggest for the benefit of science.

In the next volume we propose to publish a series of articles on Anæsthetics in which the philosophy of anæsthesia will be considered, as well as the practical details of the administration of individual agents. The latest discoveries, both theoretical and practical, will be embodied in these papers.

The monthly publication, having met with general approbation, will be continued, while it demands articles of less length for each number, it furnishes readers with more recent information than the quarterly form, and elaborate papers can always be divided and published in a series.

